# Oncolytic Probiotics with Molecular Pili for Solid Tumor Therapy

**DOI:** 10.1002/advs.202517989

**Published:** 2026-02-03

**Authors:** Haodong Ge, Chengsheng Ding, Xiao Yang, Si Gao, Changjie Yang, Yuchen Hou, Hongye Wang, Linke Bian, Hao Zhong, Yifan Qu, Luyang Zhang, Junjun Ma, Zhengwei Cai, Wenguo Cui, Minhua Zheng

**Affiliations:** ^1^ Department of General Surgery Ruijin Hospital Shanghai Jiao Tong University School of Medicine Shanghai P. R. China; ^2^ Department of General Surgery, Shanghai Key Laboratory of Gastric Neoplasms, Shanghai Institute of Digestive Surgery, Ruijin Hospital, Shanghai Jiao Tong University School of Medicine Shanghai P. R. China; ^3^ Department of Biliary‐Pancreatic Surgery Renji Hospital Affiliated to Shanghai Jiao Tong University School of Medicine Shanghai P. R. China; ^4^ Institute of Molecular Medicine (IMM) State Key Laboratory of Oncogenes and Related Genes Shanghai Cancer Institute Department of Oncology Renji Hospital School of Medicine Shanghai Jiao Tong University Shanghai P. R. China; ^5^ Department of Hepatobiliary Surgery The First Affiliated Hospital of USTC Division of Life Sciences and Medicine University of Science and Technology of China Hefei P. R. China; ^6^ Shanghai Cancer Institute State Key Laboratory of Systems Medicine for Cancer Renji Hospital Shanghai Jiao Tong University School of Medicine Shanghai P. R. China; ^7^ Department of Orthopaedics Shanghai Key Laboratory for Prevention and Treatment of Bone and Joint Diseases Shanghai Institute of Traumatology and Orthopaedics Ruijin Hospital Shanghai Jiao Tong University School of Medicine Shanghai P. R. China

**Keywords:** cell and gene therapy, mitochondrial dys‐function, oncolytic bacteria, probiotics, solid tumors

## Abstract

Cell therapy and oncolytic viruses have emerged as promising cancer treatments but face significant challenges in solid tumors due to immune suppression and gene‐related toxicities. Here, we selected a probiotic *Lactobacillus rhamnosus* (LR) that appears to exert oncolytic activity by inducing massive calcium influx, which subsequently triggers a lethal ROS burst in tumor cells. To reduce systemic toxicity and enhance oncolytic efficacy at the tumor site, we designed molecular pili (MP) targeting collagen‐rich solid tumors and modified them into LRs via chemical coupling (LR@MP). In mouse models of colorectal cancer and melanoma, LR@MP increased intratumoral accumulation by two times and enhanced bacterial clearance from peripheral tissues. At a safe dose of 4 × 10^5^ CFU, LR@MP inhibited 60%–80% of tumor growth. This dual‐optimization strategy provides a new approach for next‐generation in vivo therapies and warrants further preclinical evaluation.

## Graphical abstract

This study identifies *Lactobacillus rhamnosus* as an intrinsic oncolytic agent that triggers tumor metabolic collapse via calcium‐dependent ROS bursts. By chemically anchoring collagen‐targeting “molecular pili” to the bacterial surface, the engineered non‐transgenic probiotic (LR@MP) achieves targeted colonization and potent solid tumor suppression, validating a novel metabolically driven therapeutic paradigm distinct from traditional immunotherapies.

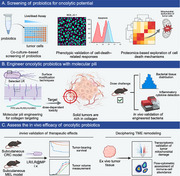

## 1 Introduction

Cancer treatment with cellular immunotherapies and oncolytic viruses (OVs) has offered many new opportunities but experiences high obstacles in solid tumors, such as the unfavorable tumor microenvironment (TME), antigen heterogeneity, and safety considerations of systemic delivery [1, 2]. To find a way around these shortcomings, bacterial‐based cancer treatment has emerged as a viable alternative. Facultative anaerobes, especially attenuated pathogens such as *Salmonella typhimurium*, bear intrinsic tumor‐targeting devices and perform oncolysis via multiple mechanisms: they generate competition with nutrients (e.g., glucose and amino acids), disrupt the tumor vasculature by inducing TNF‐ α and release pore‐forming toxins or cytolysins to directly lyse cancer cells [3, 4, 5]. Nevertheless, their application to clinical use is grossly limited by native virulence. Regular occurrence of cytokine storms, septic shock, and off target infections are also triggered by systemic administration and subsequently mandates the lowering of dosage, which ironically negatively affects the effectiveness of therapeutic colonization [3, 6]. In sharp contrast, a better safety profile (so‐called Generally Recognized As Safe or GRAS) is provided by probiotics (e.g., *E. coli Nissle 1917*, *Lactobacilli*) [7, 8, 9]. Given their inherent safety and immunomodulatory properties, probiotics—particularly *E. coli Nissle 1917 (EcN)* and the genus *Lactobacillus*—are increasingly being engineered as living foundries to restructure the immunosuppressive TME [10, 11].

Although immune stimulation (e.g., TLR/STING) is well‐documented, the bioenergetic struggle between bacteria and cancer cells has been largely neglected [12, 13, 14]. Recent data show that bacteria can radically change the tumor metabolic environment in two different ways. First, through nutrient scavenging, highly proliferative bacteria compete for glucose and essential amino acids (e.g., arginine), producing a starvation microenvironment that regulates tumor growth [3, 15, 16]. And metabolite‐mediated toxicity: certain bacterial strains release bioactive metabolites, including nitric oxide (NO) or reactive oxygen species (ROS) or short‐chain fatty acids, which may disrupt tumor mitochondrial respiration and cause oxidative stress [17]. These metabolic interactions [18, 19] are, however, short ‐lived such that there is poor bacterial retention [20]. It is on this that we postulate a speculative theory: that maximization of intratumoral retention through selected enrichment measures will not only persist in colonization, but to a complementary level of metabolic disaster termed metabolic catastrophe. We hypothesize that with the continued growth of probiotics, a localized bioenergetic kill zone is generated, in which sustained nutrient deficiency and the build up of bacterial metabolic waste products lead to permanent impairment of the mitochondrial activity of cancer cells, resulting in a distinct type of cell death that is metabolically‐mediated.

To overcome genetic engineering and endow probiotics with strong tumor‐homing functions, we designed a surface chemical display approach to the tumor extracellular matrix (ECM), the geometry which forms a widespread physical barrier in solid tumors that blocks immune cell infiltration and is associated with poor prognosis [20, 21]. We determined type I collagen to be a conserved anchoring dock. More importantly, whereas sparse tumor stroma is a physical obstacle that restricts the infiltration of conventional drugs [22, 23, 24], sparse tumor stroma is a particular therapeutic opportunity of living therapeutics. We hypothesise the ability of probiotics to anchor onto collagen fibres and use their autonomous growth ability to avoid being swept out by the hemodynamic circulation can alter the inviolable stroma into a site of colonization. In order to achieve this, we designed a decorin‐based ligand (RLRELHLNNNC) and used the Sulfo‐SMCC crosslinker to produce a sustainable conjugation with the probiotic surface. This is done by employing bacterial surface amines to the formation of stable amide bonds to form a pillar‐like structure known as the molecular pili (MP) structure. This design guarantees targeted adhesion to the tumor collagen mesh work and accumulation of deep tissue, but without interfering with the natural safety profile of the non‐transgenic bacterial chassis.

**SCHEME 1:**
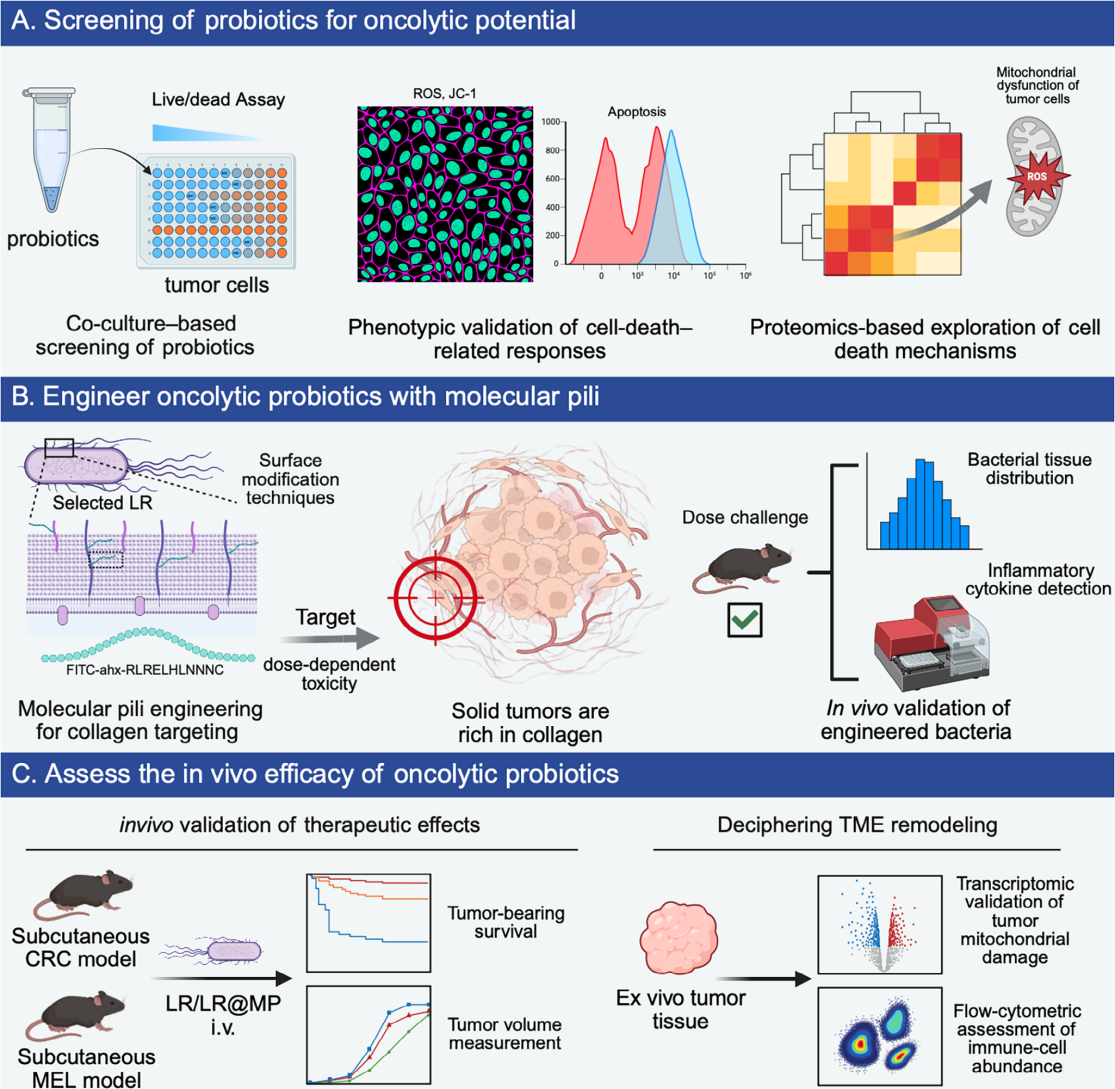
(A) Screen strains with antitumor activity from the established probiotic library; then validate phenotypes and perform proteomic analyses to elucidate the mechanisms of tumor cell death. (B) Chemically conjugate a collagen‐targeting peptide to the surface of *Lactobacillus rhamnosus* (LR) to enhance its enrichment within solid tumors; subsequently assess safety by plating bacteria recovered from isolated organs and by measuring serum inflammatory cytokines. (C) Evaluate the efficacy of LR@MP across different solid tumor models; perform RNA‐seq on excised tumors to analyze downstream mitochondrial injury; and use flow cytometry to assess how LR@MP remodels the tumor immune microenvironment.

We hypothesized that anchoring probiotics to collagen would prevent washout, allowing persistent metabolic activity to trigger a lethal ROS burst in the tumor. In order to test this hypothesis, we initially screened candidate strains and found that *Lactobacillus rhamnosus* (LR) was the best candidate with high intrinsic cytotoxicity. Then, we prepared the engineered formula, LR@MP, with the chemical display of a collagen‐targeting peptide on the LR surface. The achievement of synthesis and surface functionalization was strictly checked through flow cytometry and confocal microscopy. We then used co‐culture system, and collagen adhesion assays to confirm its particular binding affinity and increased tumoricidal activities (Scheme 1). Lastly, this targeted engineering strategy has been proven to be significantly more efficient in increasing intratumor accumulation and exerting antitumor effects in melanoma and colorectal cancer models through comprehensive in vivo evaluation and through the proposed metabolic mechanism.

## 2 Results

### 2.1 Oncolytic Probiotic LR Induces Tumor Cell Death via Mitochondrial ROS Burst

**FIGURE 1:**
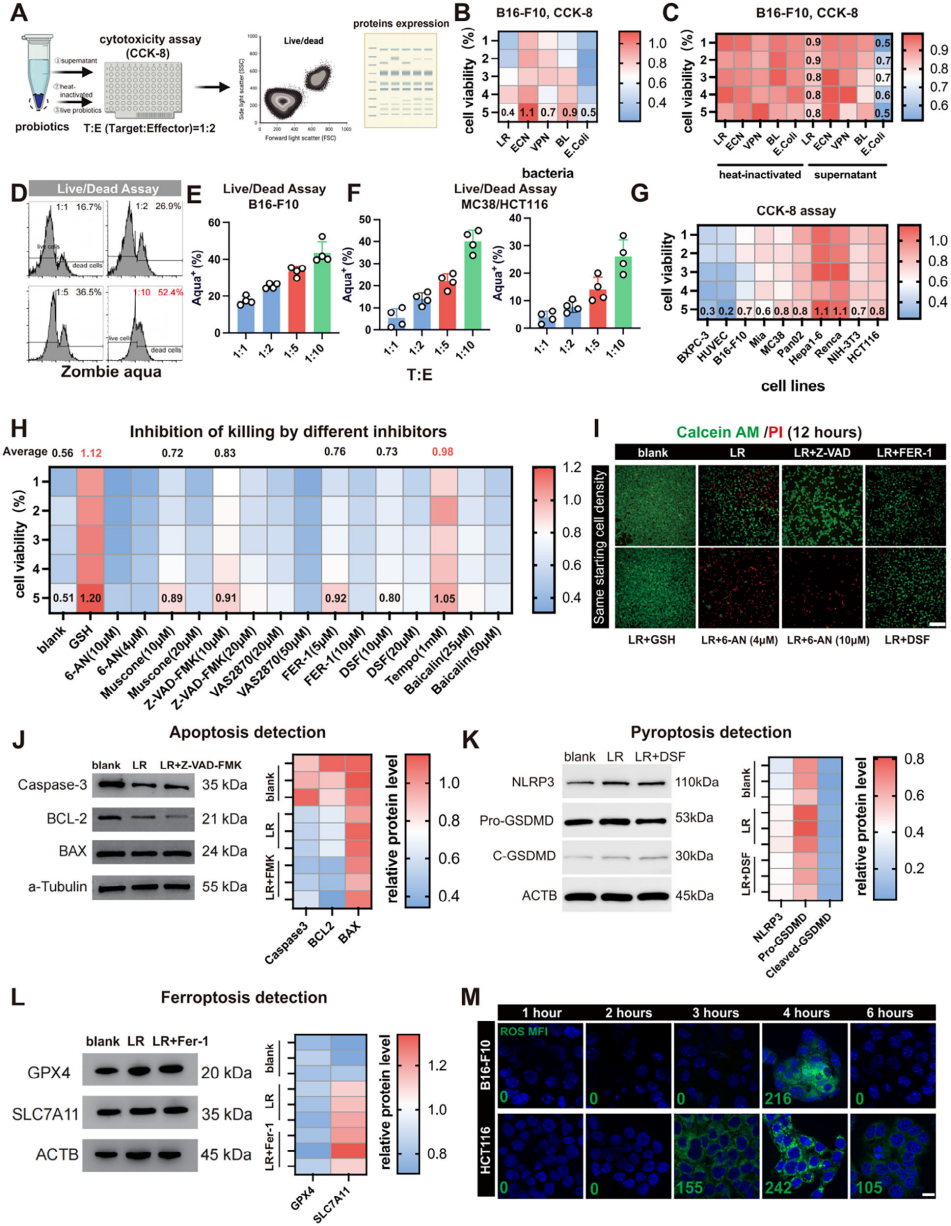
LR induced tumor cell death via mtROS burst. (A) Co‐culture experiment protocol. (B) Cell toxicity of different bacteria (*n* = 5). ECN, *Escherichia coli Nissle 1917*; VPN, *VPN20009*; BL, *Bifidobacterium longum; E. coli*, *Escherichia coli*. (C) Co‐culture of heat‐inactivated bacteria and bacterial supernatant with tumor (*n* = 5). (D–F) Live/dead flow cytometry and data analysis (*n* = 4). (G) Toxicity of LR to different cell lines (*n* = 5). (H) Inhibition efficiency of different death related inçibitors (*n* = 5). (I) Further confirmation of LR‐induced cell death. Scale bar, 200 µm. (J–L) Verification of apoptosis, pyroptosis, and ferroptosis proteins, representative Western blotting images (*n* = 3). (M) Representative confocal images for ROS detection. Scale bar, 40 µm. The MFI was analyzed using ImageJ software.

The cell viability and protein expression of tumor cells were studied by coculture of various common probiotics, their metabolites, and attenuated strains with B16 ‐ F10 cells (Figure 1A). LR showed strong tumor cell cytotoxicity among them, but *E. coli*, which was used as non‐probiotic control showed too strong toxicity (Figure 1B). We first avoided the cytotoxicity of Lactobacillus rhamnosus (LR) cell‐free supernatant and heat‐inactivated bacteria, which further confirmed that the oncolytic activity of this microorganism depends on its viability (Figure 1C). Live/Dead staining and CCK‐8 confirmed that LR has generalized cytotoxic effects on a collection of solid tumor cells. It is worth noting that low concentrations of LR caused more than 50 % death in B16‐F10 melanoma cells when a cell:bacteria ratio was 1:10 (Figure 1D–G). In order to understand the underlying mechanism, we performed a broad pharmacological screen to identify inhibitors of apoptosis, ferroptosis, and pyroptosis. Interestingly, this was a pronounced cytotoxicity that was heavily linked to the reactive oxygen species (ROS), specifically, the mitochondrial reactive oxygen species (mtROS). Analysis of hallmark proteins by immunoblotting showed a complicated phenotype with the simultaneous participation of various cell death pathways (Figure 1H–L). We then confirmed that LR treatment provokes the formation of a strong mitochondrial accumulation of ROS (Figures 1M; S1A). Mechanistically, B16‐F10 cells silenced with siRNA targeting the oxidoreductase p66Shc strongly inhibited LR‐induced the generation of mitochondrial superoxide (measured by MitoSOX) and improved the survival of the cells (Figure S1B–E) [25].

### 2.2 LR Induces ROS Burst by Triggering Calcium Overload

**FIGURE 2:**
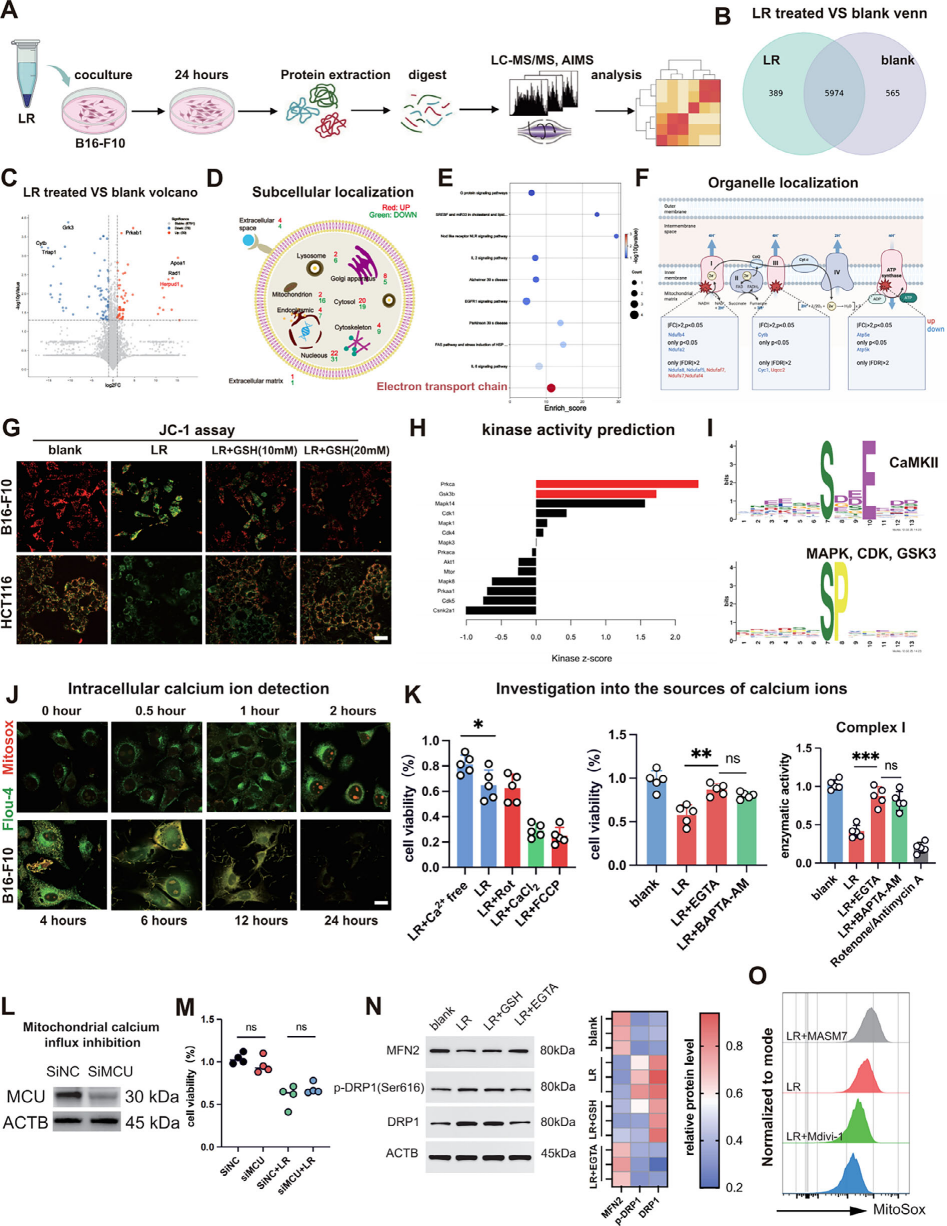
LR induces mitochondrial dysfunction in tumor cells. (A) Proteomics and phosphoproteomics workflow for detecting tumor proteins (*n* = 3). (B) Venn diagram of proteomics profiling. (C) Proteomics volcano plot (*n* = 3). (D) Subcellular localization in proteomics analysis. (E) WikiPathway enrichment analysis of proteomics results. (F) WikiPathway enrichment analysis of proteomics results. (G) Representative JC‐1 confocal images, experimental duration: 12 h. Scale bar, 40 µm. (H) Phosphoproteomics kinase prediction analysis. (I) Motif analysis of phosphorylated proteomics. (J) Representative images of calcium ion confocal microscopy. Green fluorescence represents calcium ions, and red fluorescence represents mt‐ROS. Scale bar, 25 µm. (K) Investigation into the source of calcium ions, calcium ion chelation experiment, and mitochondrial complex I enzyme activity assay (*n* = 5). (L,M) SiRNA knockout efficiency verification and cytotoxicity assay. (N) Representative Western blot images for mitochondrial fission verification (*n* = 3). (O) Mitochondrial ROS inhibition experiment, representative flow cytometry images (*n* = 3).

We have done proteomic and phosphoproteomic analysis of LR‐treated B16‐F10 cells (Figure 2A). The analysis of the Venn diagram showed that LR had only small changes in the proteome (Figure 2B). The analysis of the differential expression showed that proteins at the top of the ranking list were mainly linked to redox homeostasis (Figure 2C). Subcellular localization profiling also revealed a broad downregulation of the mitochondrial proteome (Figure 2D). WikiPathways enrichment also particularly contributed to the identification of the electron transport chain (ETC), and on closer look the selective silencing of subunits in the Complex I and III (Figure 2E,F). In line with such results, the JC‐1 staining revealed LR‐induced depolarization of mitochondrial membrane potential across different tumor cell lines (Figure 2G). In one direction, the prediction of kinase activity was protein kinase (PKC), which closely interacts with calcium (Figure 2H). Analysis of phosphorylation motifs shows that the most upregulated motifs are related to MAPK and CaMKII, and are also strongly related to calcium signaling (Figure 2I). As a result, we observed the intracellular calcium changes and found that there was an increase in cytosolic calcium levels with time. Mechanistic interventions that required calcium‐free media and calcium replenishment showed that the cytotoxicity of LR is dependent on the extracellular influx of calcium (Figures 2K; S1F). In fact, it was determined that pretreatment with either the extracellular calcium chelator EGTA or the intracellular one BAPTA‐AM effectively rescued B16‐F10 cells from death, with no significant difference observed between the two (Figure 2K). More importantly, the inhibition of the enzymatic activities of Complex I and III by LR was inhibited by the EGTA chelation (Figures 2K; S1F). Nonetheless, MCU knockdown did not result into any toxicity resistance in B16F10 cells (Figure 2L,M). Since cytoplasm calcium overload is reported to induce fission‐dependent ROS bursts, we studied the dynamics of mitochondria and found a transition to fission, which was manifested by increased fission and decreased fusion indicators (Figure 2N) [26]. Mdivi‐1‐inhibited mitochondrial fission with removal of mitochondrial ROS creation and MASM7‐prompted fission with cell death enhanced mitochondrial cytotoxicity was greatly reduced by pharmacological fission inhibition using Mdivi‐1 (Figures 2O; S1G,I).

### 2.3 Engineering LR@MP for Tumor‐Targeted Accumulation and Off‐Tumor Clearance

Our preliminary data indicated that LR cytotoxicity is dose‐dependent and synergistically enhanced by glycolysis inhibition (e.g., 2‐DG) (Figures 1E; S2A). However, in vivo dose‐escalation revealed a narrow therapeutic window: while 4 × 10^5^ CFU caused transient weight loss, increasing the load to 1 × 10^6^ CFU or combining with 2‐DG triggered lethal acute hepatitis (Figure S2B,C). To dissociate systemic hepatotoxicity from antitumor efficacy, we targeted type I collagen (COL1A1), a ubiquitous ECM component associated with poor prognosis across solid tumors (Figure S2D–H) [27].

**FIGURE 3:**
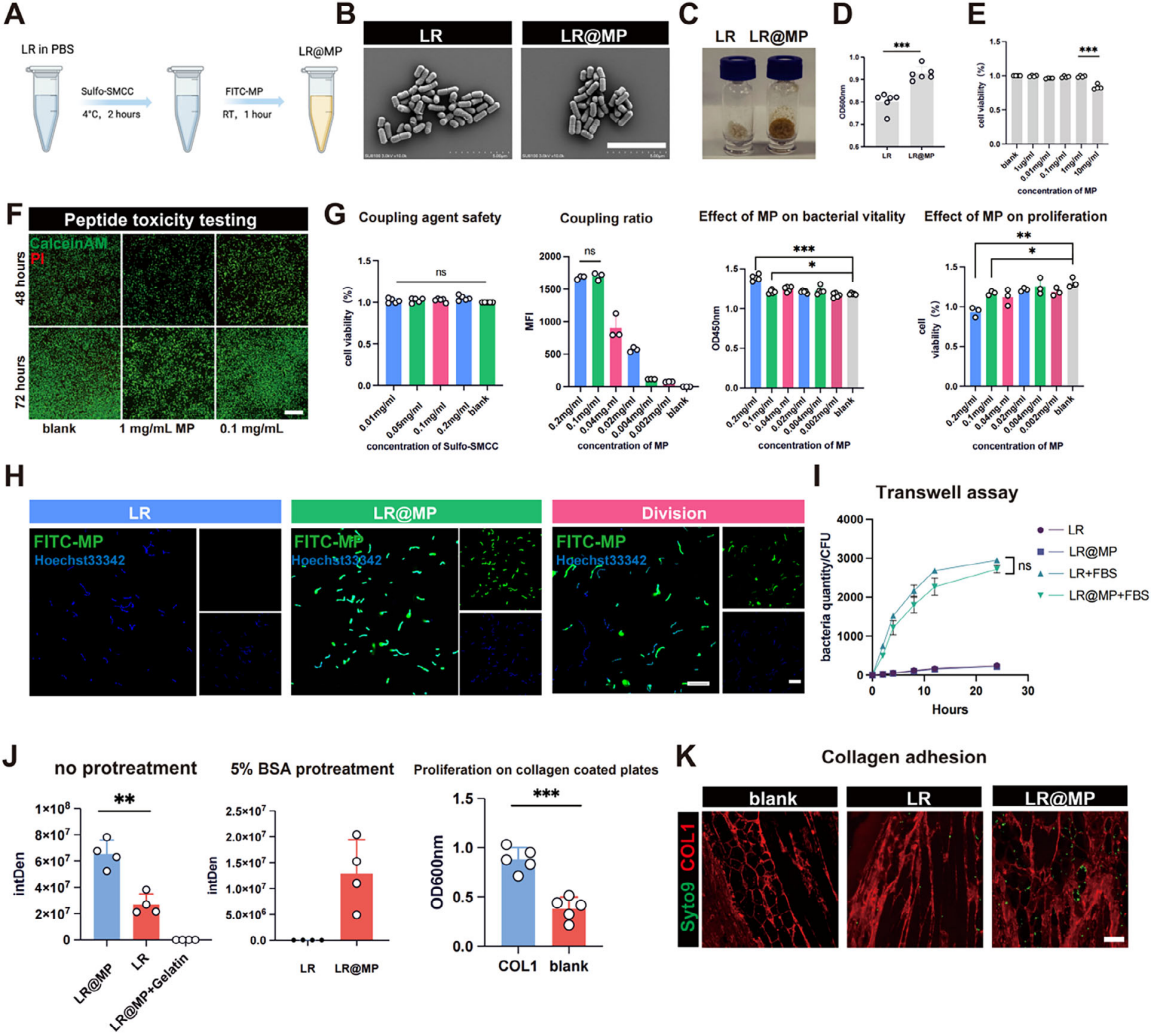
Construction and characterization of molecular pili‐engineered probiotics. (A) Preparation of LR@MP. (B) SEM images of LR and LR@MP. Scale bar, 5 µm. (C,D) Lyophilized morphology and post‐rehydration viability of LR and LR@MP (*n* = 6). (E,F) Representative images of MP‐mediated suppression of MC38 proliferation. Scale bar, 200 µm. (G) Viability of LR treated with varying concentrations of Sulfo‐SMCC (*n* = 5); mean fluorescence intensity (MFI) of LR@MP at different modification efficiencies (*n* = 3); viability and proliferation of LR@MP with graded modification rates (*n* = 5). (H) Representative confocal microscopy images of LR@MP. Scale bar, 20 µm. (I) Transwell experiments of LR and LR@MP, with or without FBS in the basement (*n* = 3). (J) Fluorescence intensity of collagen‐coated plates incubated with LR@MP or LR (*n* = 3); proliferative capacity post‐collagen adhesion (*n* = 5). (K) LR@MP/LR localization on collagen‐coated cell culture slides. Scale bar, 25 µm.

We synthesized LR@MP via Sulfo‐SMCC conjugation (Figure 3A). TEM showed that the modification did not alter the native morphology of LR (Figure 3B), whereas greatly enhancing the recovery of LR in the presence of lyophilization (Figure 3C,D). The optimization experiment determined that 0.1 mg mL ‐1 MP and 0.2 mg mL ‐1 crosslinker were saturating without any effect on bacterial viability or host cell safety (Figure 3E,F). Transcriptomic profiling showed that MP modification increased the activity of stress‐response genes, in particular, cspA (associated with cryotolerance) and cell wall constituents (increasing immune recognition) (Figure S3A–D). LR@MP elicited superior innate immune activation compared to unmodified LR, characterized by enhanced M1 macrophage polarization (53.1% vs. 37.7%, *p* < 0.05) and dendritic cell maturation (Figure S3E–G), which correlated with increased phagocytic efficiency (Figure S3H). Macrophages exhibited greater phagocytic ability of LR@MP and were more effective in clearance in normal tissues (Figure S3I). We also found that surface‐bound FITC‐MP was diluted during bacterial division (Figure 3H). Longitudinal flow cytometry was used to show that although Mean Fluorescence Intensity (MFI) decreased over time, the high‐density bacterial population that are characterized by limited proliferation could maintain high targeting efficiency during long periods of time (Figure S3K,L). Additionally, Transwells ensured that MP modification did not have a significant negative effect on bacterial motility (Figures 3I; S3J). Crucially, LR@MP demonstrated specific, high‐affinity binding to type I collagen (> 200‐fold increase on BSA‐blocked substrates), validating its potential for tumor‐specific colonization (Figure 3J,K).

### 2.4 LR@MP Suppresses Solid Tumor Progression In Vivo via Mitochondrial‐Dysfunction

**FIGURE 4:**
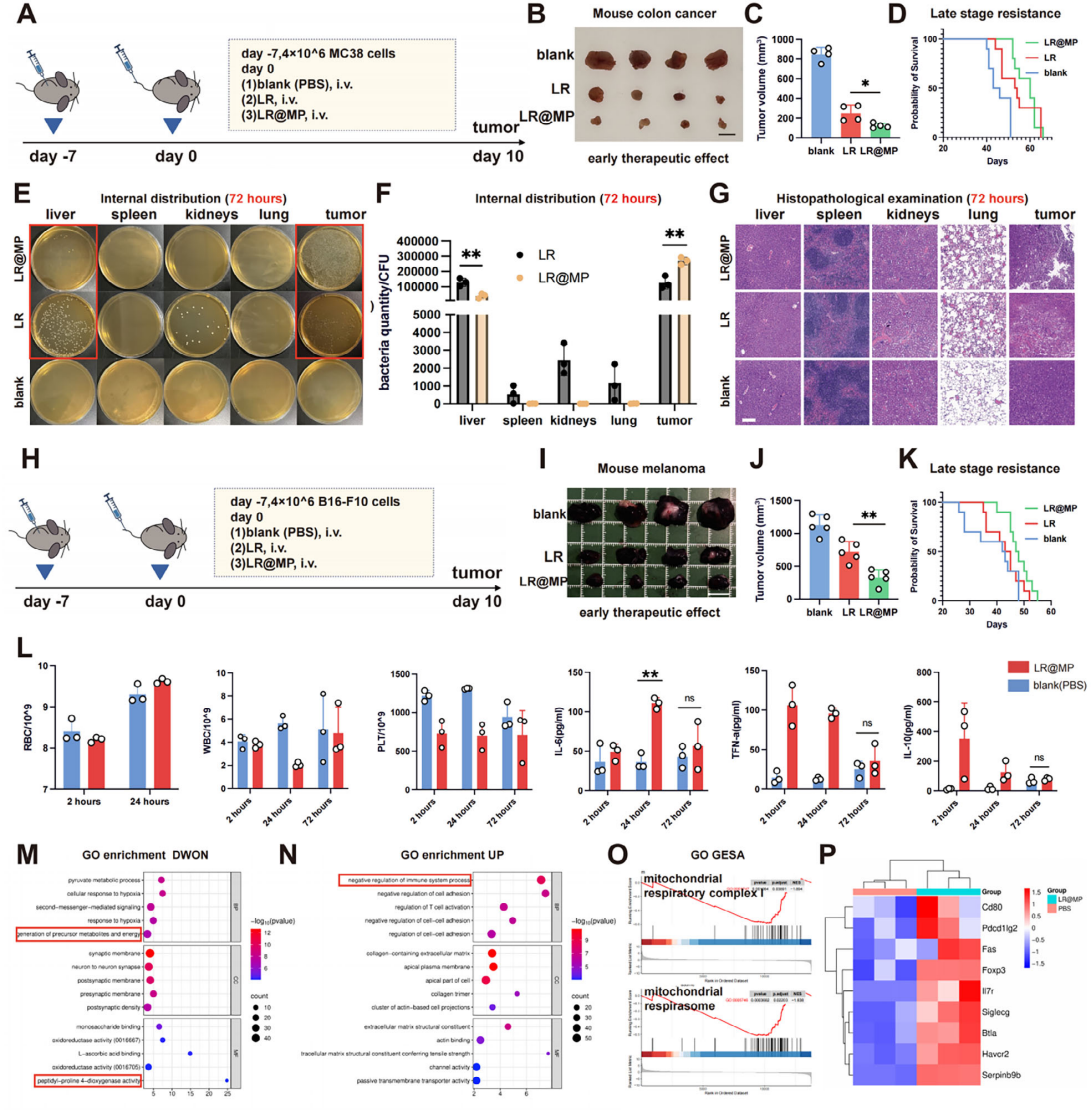
LR@MP mediates early‐stage suppression of solid tumor progression via mitochondrial‐dysfunction. (A) Animal experimental protocol. (B,C) Murine colon cancer model and tumor volume analysis (*n* = 4). Scale bar, 1 cm. (D) Survival curve of murine colon cancer model (*n* = 10). (E–G) Representative images and statistical analysis of bacterial distribution and pathological examination at 72 h post‐inoculation (*n* = 3). Scale bar, 150 µm. (H) Animal experimental protocol for the melanoma model. (I,J) Murine melanoma model and tumor volume analysis (*n* = 4). Scale bar, 1 cm. (K) Survival curve of murine melanoma model (*n* = 10). (L) Blood cell counts and circulating cytokine levels in mice post‐treatment (*n* = 3). (M,N) GO enrichment analysis of transcriptomes from LR@MP‐treated tumors vs. control tumors at 72 h post‐treatment (*n* = 3). (O) GSEA analysis of transcriptomes from LR@MP‐treated tumors vs. control tumors at 72 h post‐treatment (*n* = 3). P, Heatmap of differentially expressed genes associated with negative regulation of immune response (*n* = 3).

In colorectal and melanoma models, LR@MP demonstrated superior tumor suppression and reduced acute mortality compared to LR, although long‐term survival benefits were limited (Figure 4A–D,H–K). Biodistribution analysis at 72 h confirmed enhanced intratumoral accumulation and reduced hepatic burden for LR@MP, with transient systemic inflammation resolving within this window (Figure 4E–G,L). Immunofluorescence revealed persistent CD4^+^ and CD8^+^ T cell infiltration (Figure S3M). Mechanistically, transcriptomic profiling of treated tumors corroborated our in vitro findings: downregulated energy metabolism and mitochondrial gene sets (Figure 4M,O) aligned with significant enrichment of calcium signaling pathways (*p* < 0.0001). Concurrently, KEGG Enrichment Analysis revealed a significant upregulation of immunosuppressive signatures, specifically those governing negative T‐cell regulation.

### 2.5 MHC1‐High and PD‐L1‐High Senescence Tumor Cells Escape LR‐Mediated Killing via Metabolic Reprogramming

**FIGURE 5:**
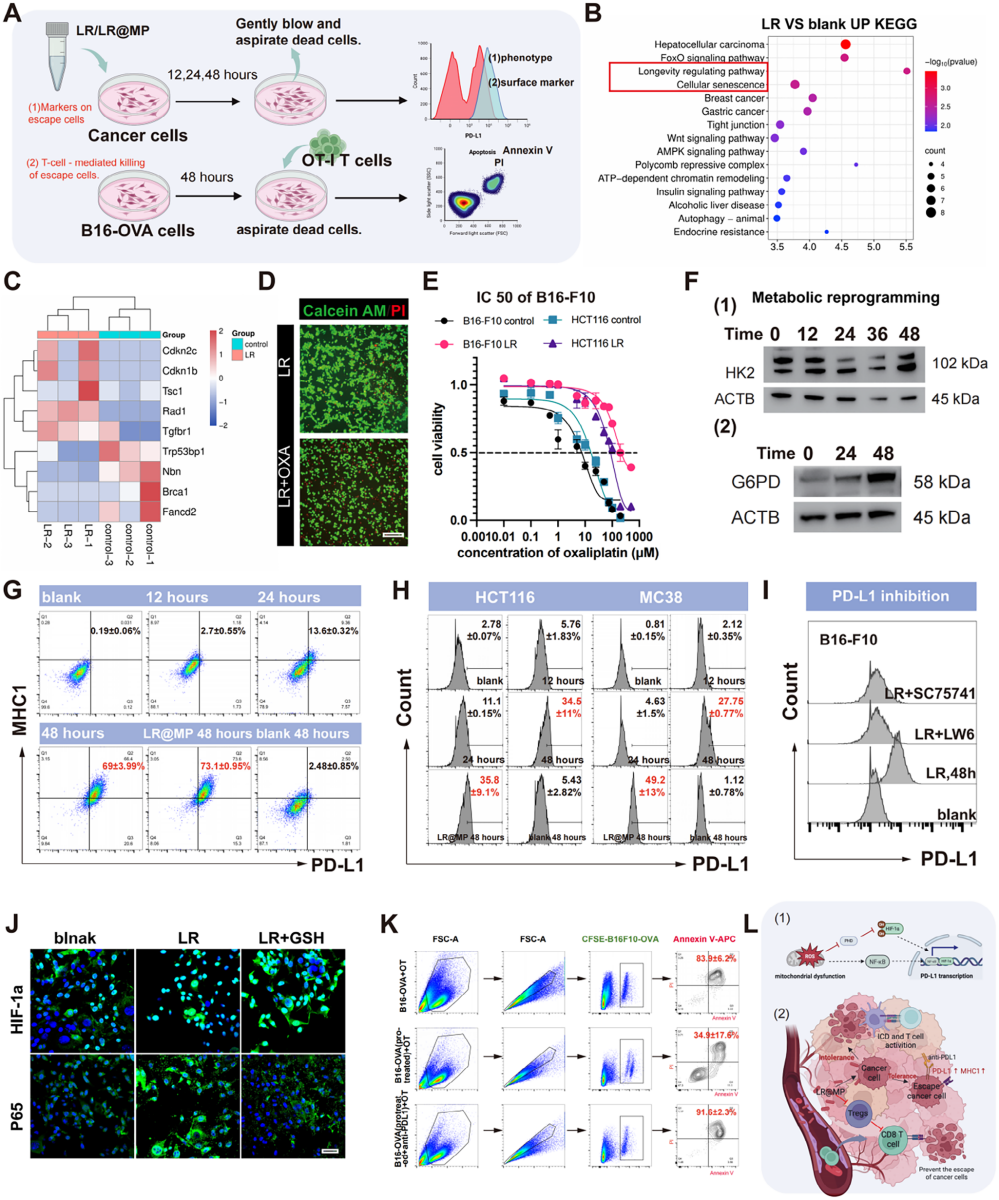
Senescent tumor cells escape LR‐mediated killing via metabolic reprogramming. (A) Screening of probiotics for eliminating escaped tumor cells via phenotypic and surface marker characterization. (B) Integrated phosphoproteomic and proteomic analysis with KEGG enrichment of downregulated phosphosites and upregulated proteins. (C) Heatmap of senescence‐associated protein expression changes (*n* = 3). (D) Live/dead cell staining. Scale bar, 200 µm. (E) Oxaliplatin IC50 values (*n* = 3). (F) Representative Western blot images of metabolic reprogramming‐related proteins. (G) Representative flow cytometry plots of surface molecule expression in B16‐F10 cells (*n* = 3). (H) Representative images of PD‐L1 expression in other cell lines (*n* = 3). (I) Effects of transcription factor inhibitors on PD‐L1 expression. (J) Enhanced nuclear translocation of HIF‐1α and NF‐κB. (K) Cytotoxicity assay of probiotics against OT‐1 and B16‐OVA tumors at 10:1 effector‐to‐target ratio (*n* = 3). (L) Proposed mechanism of probiotic‐induced tumor immune escape.

In order to study the phenomenon of immunosuppression, we have created LR‐selected survivor lines and defined their phenotypes (Figure 5A). Basal phosphoproteomics analysis showed the enrichment of senescence‐related pathways (Figure 5B,C). Since senescence is associated with multidrug resistance [28], the chemosensitivity was assessed. Although LR itself had no effect on the activity of the oxaliplatin (OXA) (Figure 5D), the IC50 value of OXA in LR‐pretreated B16‐F10 and HCT116 cells rose significantly (Figure 5E). This metabolic resistance was associated with mitochondrial damage: whereas Hexokinase 2 (HK2) initially went down (Figure 4M, in line with mitochondrial damage), it later recovered together with gradual G6PD upregulation (Figure 5F). Improved G6PD activity leads to the clearance of ROS and repair of DNA, as a result, oxidative stress and damage caused by chemotherapy are reduced (Figure 5F).

As our transcriptomic data involved T‐cell suppression (Figure 4P), we profiled immune markers. In cells treated to LR, temporary upregulation of MHC‐I was observed and then significantly PD‐L1 upregulation after 24 h, which was enhanced by LR@MP (Figure 5G,H). Mechanistically NF‐ kB and HIF‐1 nuclear translocation inhibitors had a great suppressive effect on this PD‐L1 upregulation (Figure 5I). Confocal microscopy demonstrated elevated nuclear signal of p65 and HIF‐1α (Figure 5J), which indicates that protracted PD‐L1 is provoked by the activation of these transcription factors by mitochondrial ROS. Lastly, T‐cell cytotoxicity was determined. Although B16‐OVA cells that had been treated with LR avoided OT‐1 T cell killing, blockade of PD‐L1 reinstated and significantly increased cytotoxic effectiveness exceeding that of control (Figure 5K).

### 2.6 Targeting PD‐L1 to Eliminate Senescent Tumor Cells Stabilizes Long‐Term Efficacy of LR@MP

**FIGURE 6:**
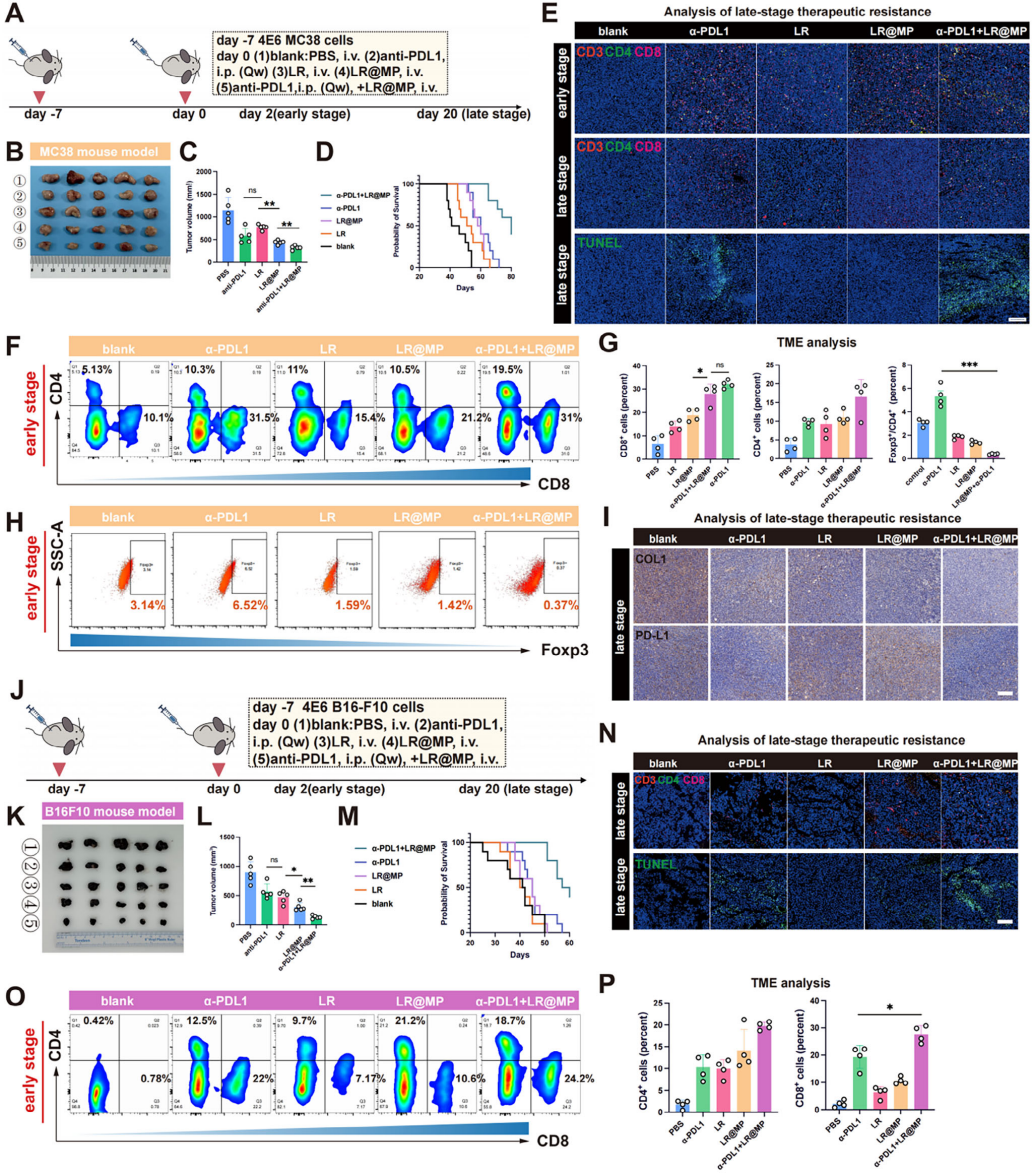
Long‐term therapeutic effect of LR@MP and its efficacy on primary tumors. (A) Animal experiment protocol. The dose of anti‐PD‐L1 is 100 µg. (B,C) Mouse colon cancer and tumor volume analysis (*n* = 5). (D) Survival curve of mouse colon cancer (*n* = 10). (E) Representative images of tumor T cell IF. Scale bar, 150 µm. (F–H) Post‐treatment T cell and Treg infiltration in tumors and statistical analysis of data (*n* = 4). (I) Representative IHC images of PD‐L1 and COL1 in late‐stage treatment. Scale bar, 150 µm. (J) Animal experiment protocol. The dose of anti‐PD‐L1 is 100 µg. (K,L) Mouse melanoma cancer and tumor volume analysis (*n* = 5). (M) Survival curve of mouse melanoma cancer (*n* = 10). (N) Representative images of tumor T cell IF. Scale bar, 150 µm. (O,P) Post‐treatment T cell in tumors and statistical analysis of data (*n* = 4).

Given that the upstream drivers of this resistance (NF‐κB and HIF‐1 signaling) are also essential for the activation and survival of effector T cells, employing broad‐spectrum pathway inhibitors risks inadvertently dampening the anti‐tumor immune response [29]. Therefore, to specifically target the resistant tumor cells without compromising T‐cell viability, we prioritized immune checkpoint blockade (anti‐PD‐L1 antibody) over small‐molecule inhibitors. We continued to validate our findings in mouse models of colorectal cancer and melanoma (Figure 6A). In both colorectal cancer and melanoma animal models, the combination with anti‐PD‐L1 showed the best tumor suppression efficiency (Figure 6B,C,K,L). More importantly, the combination with anti‐PD‐L1 significantly extended the median survival time of mice (Figure 6D,M). During early treatment, we found that LR, LR@MP, and the combination group all significantly enhanced the infiltration of CD4+ and CD8+ T cells, with the combination group recruiting the most of both T cell types (Figure 6E–G,O,P). Meanwhile, we observed that anti‐PD‐L1 monotherapy resulted in a higher proportion of regulatory T cells (Tregs), thereby inhibiting T cell cytotoxicity. In contrast, the combination group significantly reduced the Treg proportion (Figure 6G,H). In late‐stage treatment, TUNEL staining revealed that probiotics alone lost their tumor‐killing capacity, while the combination group maintained probiotic‐mediated tumor killing (Figure 6E). Additionally, immunohistochemistry showed that LR@MP and combination treatments significantly reduced collagen content (Figure 6I), since MP can not only adhere to collagen but also interfere with collagen remodeling [27]. Concurrently, probiotic treatment markedly increased PD‐L1 expression, while combination with anti‐PD‐L1 suppressed this immunosuppressive molecule and promoted clearance of escaping cells (Figure 6I).

## 3 Discussion

### 3.1 Delivery Chassis Changed into Intrinsic Killer

Most of the current bacterial therapeutics are based on a “delivery chassis” model, such as *E. coli Nissle 1917* (EcN), which uses genetic engineering to secrete therapeutic vectors (e.g., cytokines, nanobodies). Though successful, the method poses certain difficulties related to GMO laws and the stability of the payload. Conversely, we have determined *L. rhamnosus* (LR) to be an effective oncolytic agent. In contrast to EcN, which in most cases is an inert subvector that necessitates high doses (10^8^ CFU) of the bacterium, LR exhibits strong cytotoxicity at low doses (10^5^ CFU) by a unique metabolic action [10, 11, 12, 13, 14].

### 3.2 Mechanism of Metabolically Programmed Cytotoxicity

One important discovery in this study is the Calcium‐ROS‐Bioenergetic″ axis that was explained. Our hypothesis is to suggest that it is a generalizable occurrence amongst high‐acid‐producing probiotics and that this metabolically programmed cytotoxicity is optimized in LR because of its specific metabolic program. LR releases fatty acids of short chains (SCFA) and a lactic acid cocktail of high concentration. We can support our mechanistic data and literature that lactate acidifies the microenvironment to activate Acid‐Sensing Ion Channels (ASICs), whereas SCFAs (acetate/propionate) activate GPR43 receptors to stimulate Gq‐mediated calcium release [30, 31]. The resulting huge extracellular calcium influx overloads mitochondria, initiating fission (Drp1 activation) and collapsing the electron transport chain (Complex I/III), with the final result of releasing a fatal burst of ROS. It is a different mechanism of metabolic siege, independent of an immune‐based mechanism of *Salmonella* or *Listeria* [30, 31, 32] mechanisms.

### 3.3 Rational Engineering

We utilized a chemical conjugation technique to present Collagen‐binding Molecular Pili (MP), which is a selection based on clinical pragmatism. Chemical modification, unlike genetic engineering, which is frequently undermined by the inherent instability of the plasmid vectors, which tend to lose segregation when they are subjected to the normal environment in the complex real world without antibiotic pressure, and it has the inherent biosafety risk of horizontal gene transfer to the resident microbiome.

One of the frequent criticisms of chemical modification is that surface moieties are affected by the dilution of surface moieties during cell division. Nevertheless, a Two‐Stage Colonization model is presented in our kinetic analysis (Figure S3) and in vivo data. Only the first phase of the MP coating (0 ‐ 6 h) is essential to try to prevent the washout of the bacteria by the hemodynamic action [33, 34]. After being anchored, the bacteria multiply and lose the MP coat, but at this point, the bacteria will have been located in the hypoxic/immunosuppressive niche, and the additional targeting moieties will not be required. Although chemical conjugation was a strong demonstration of proof‐of‐concept, these data give direct motivation to sophisticated approaches to genetic engineering in the improvement of this platform. Considering that the lactate/SCFA‐calcium axis is the central fatal pathway we identified, future strains can be programmed to reroute endogenous metabolic fluxes, e.g., by placing strong promoters at the start of the lactate dehydrogenase (ldh) or pyruvate oxidase (pox) genes. This would enable bacteria to autonomously release greater amounts of oncolytic metabolites, which would escalate tumor bioenergetic breakdown without the need to inject exogenous protein cargos. Also, to overcome surface moiety dilution permanently, the validated collagen‐binding domain might be genetically linked to bacterial outer membrane proteins (e.g., OmpA) or pili subunits. This would result in a self‐treating targeting interface that persists during cell division, which will guarantee a long‐term survival even in high growth rate.

### 3.4 Operation of Resistance and Combined Immunotherapy

Although early‐stage tumor suppression of the immune system and massive infiltrations of the CD4 + and CD8 + T cells (Figure 5G) were seen, both LR and LR@MP therapies failed to translate into a sustained survival benefit (Figure 5D,K). Transcriptomic analysis revealed the mechanism of this discrepancy, at early cytokine periods, there was an observed immunomodulators response, but at late‐stage tumors, there was an observed strong upregulation of immunosuppressive pathways, in particular, capable of inhibiting T‐cell (Figure 5N,P). We describe this as a ROS system adaptive resistance program. In metabolic stress, residual tumor cells increase G6PD to eliminate ROS, besides amplifying the expression of PD‐L1 using the ROS‐HIF‐1‐NF‐KB pathway. This adaptation has a special vulnerability: the survival cells get metabolically stable, but expose themselves to the immune system. This result can help understand the rationality behind the use of LR@MP in combination with anti‐PD‐L1 antibodies. Under this strategy, the bacteria would transform the cold tumors into the hot ones through the immunogenic cell death, and the antibody would release the definite checkpoint brake laid on the immune attack by such a tumor, which would further maintain the assault.

### 3.5 Safety Profile and Therapeutic Efficacy

We admit Maximum Tolerated Dose (MTD) of LR (4 × 10^5^ CFU) is lower than the attenuated strains of EcN (10^8^ CFU). This is, however, an indicator of a better Therapeutic Index. In many cases, the ecN takes large amounts of doses to be colonized, making genetic attenuation (like ΔmsbB) necessary in this case to avoid septic shock [5]. Conversely, LR@MP exhibits high tumor regression with a low bio‐burden with its specific retention and strong metabolic toxicity. Moreover, the increased TNF‐a noted (Figure 5L) is a positive cytokine warning signal that bypasses localized immunosuppression without inducing systemic toxicity. Coupled with the fact that LR is a Gram‐positive probiotic devoid of LPS (endotoxin), this means that the risk of endotoxin‐mediated shock is not incurred, as well as the fact that the inflammatory response is temporary and can be treated with ease [35, 36].

### 3.6 Restrictions and Future Prospect

The existing shortcomings are that the gradients utilized are intratumoral/peritumoral that could not be similar in every patient. The future work is aimed at: (1) Testing the kind of hypothesis of the Metabolic Kill Zone in patient‐derived xenografts (PDX); (2) Developing genetic approaches to stably express collagen‐binding peptides for sustained targeting; and (3) The exploration of the systemic immune memory elicited by this metabolically enhanced oncolysis to prevent metastasis.

## 4 Conclusion

This work validates a non‐transgenic approach that overcomes the challenge of balancing tumor accumulation with safety, effectively repurposing standard probiotics for cancer therapy. Our findings suggest that future live biotherapeutics should prioritize maximizing metabolic toxicity to exploit the metabolic link between bacteria and tumors. However, a key limitation remains: prolonged metabolic stress drives adaptive resistance in residual tumor cells, specifically through PD‐L1 upregulation. Consequently, clinical translation cannot rely on monotherapy. Combining metabolically active bacteria with immune checkpoint blockade is essential to overcome this resistance and achieve durable remission.

## Conflicts of Interest

The authors declare no conflicts of interest.

## Supporting information




**Supporting File 1**: advs74120‐sup‐0001‐SuppMat.docx

## Data Availability

The data that support the findings of this study are available from the corresponding author upon reasonable request.
