# Supplementary material for: Oncolytic Probiotics with Molecular Pili for Solid Tumor Therapy
Source: Adv Sci (Weinh). 2026 Feb 3;13(19):e17989. doi: 10.1002/advs.202517989 (PMC13045323; doi:10.1002/advs.202517989)
Supplement: Supplementary file 1 — Supporting File 1: advs74120‐sup‐0001‐SuppMat.docx [file ADVS-13-e17989-s001.docx]

**Oncolytic Probiotics with Molecular Pili for Solid Tumor Therapy**

*Haodong Ge, Chengsheng Ding, Xiao Yang, Si Gao, Changjie Yang, Yuchen Hou, Hongye Wang, Linke Bian, Hao Zhong, Yifan Qu, Luyang Zhang, Junjun Ma, Zhengwei Cai*, Wenguo Cui*, Minhua Zheng**

H. Ge, C. Ding, X. Yang, H. Zhong, L. Zhang, J. Ma, M. Zheng

Department of General Surgery, Ruijin Hospital, Shanghai Jiao Tong University School of Medicine, 197 Ruijin 2nd Road, Shanghai 200025, P.R. China.

Shanghai Minimally Invasive Surgery Center, 197 Ruijin 2nd Road, Shanghai 200025, P.R. China.

1. mail: zmhtiger@yeah.net (M. Zheng)
2. Qu, Z. Cai, W. Cui

Department of Orthopaedics, Shanghai Key Laboratory for Prevention and Treatment of Bone and Joint Diseases, Shanghai Institute of Traumatology and Orthopaedics, Ruijin Hospital, Shanghai Jiao Tong University School of Medicine,197 Ruijin 2nd Road, Shanghai 200025, P.R. China.

E-mail: caizhengwei@shsmu.edu.cn (Z. Cai), wgcui@sjtu.edu.cn (W. Cui)

1. Gao

Department of Clinical Laboratory Medicine, Shanghai Chest Hospital, School of Medicine, Shanghai Jiao Tong University, Shanghai 200025, P.R. China.

1. Yang

Institute of Molecular Medicine (IMM), State Key Laboratory of Oncogenes and Related Genes, Shanghai Cancer Institute, Department of Oncology, Renji Hospital, School of Medicine, Shanghai Jiao Tong University, Shanghai 200025, China.

1. Hou

Department of Hepatobiliary Surgery, The First Affiliated Hospital of USTC, Division of Life Sciences and Medicine, University of Science and Technology of China, Hefei, 230001, China.

1. Wang, L. Bian

Shanghai Cancer Institute, State Key Laboratory of Systems Medicine for Cancer, Renji Hospital, Shanghai Jiao Tong University School of Medicine, Shanghai 200025, China.

*Address correspondence to: <caizhengwei@shsmu.edu.cn> (Z. Cai), [wgcui80@hotmail.com](mailto:wgcui80@hotmail.com) (W. Cui), <zmhtiger@yeah.net> (M. Zheng)

**Keywords:** cell and gene therapy; solid tumors; probiotics; oncolytic bacteria; mitochondrial dys-function;

**1.Figures**
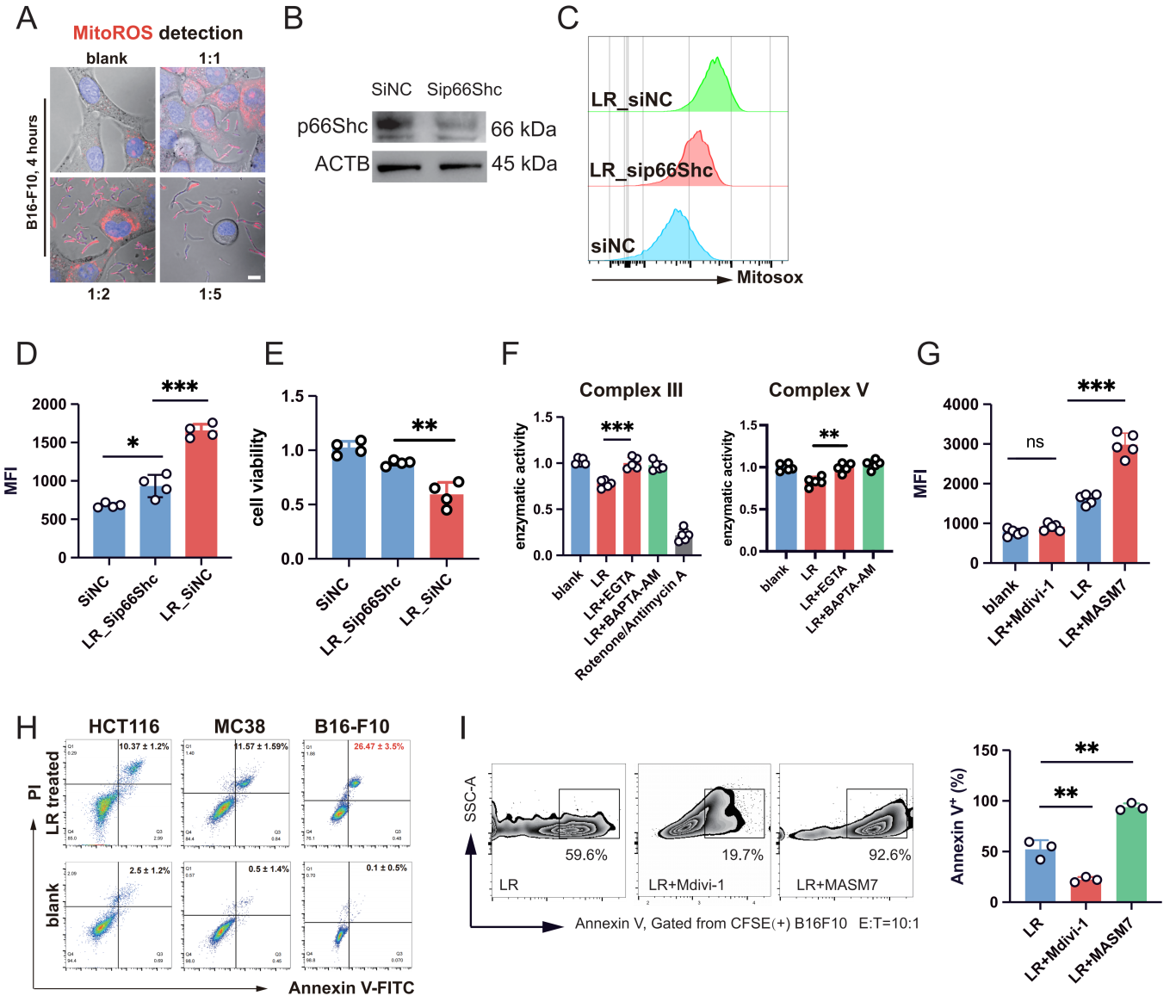


**Figure. S1** LR induced tumor cell death via mtROS burst. **A,** Representative confocal images for mt-ROS detection. Scale bar, 8 μm. **B**, SiRNA knockdown efficiency verification. **C, D**, Representative flow cytometry images from mitosox and statistics analysis (n=3). **E**, Cytotoxicity assay (CCK8, n=4). **F,** mitochondrial Complex III and Complex V enzyme activity assay (n=5). **G**, Quantitative analysis of mitochondrial ROS levels (n=5). **H**, Apoptosis detection in different cell lines (n=3). **I,** Representative flow cytometry images of LR killing experiments and statistics analysis (n=3).


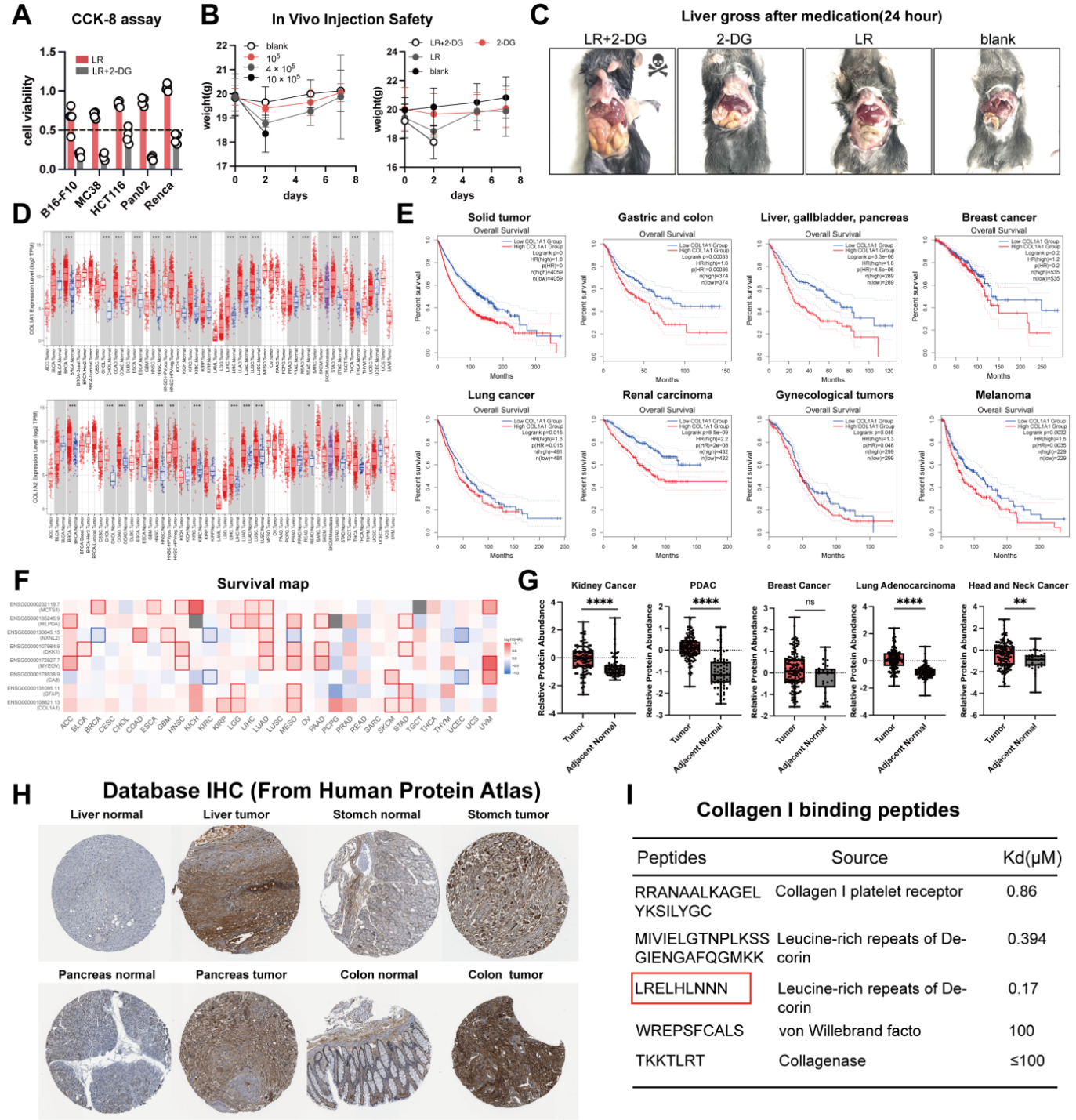


**Figure. S2** The value of type I collagen in pan - cancer research. **A**, Cytotoxicity assay of LR combined with 2-DG (n = 4). **B**, Body weight after in vivo injection (n = 3). Dose screening and toxicity tests were conducted in two separate animal cohorts. **C**, LR combined with 2-DG caused mouse death and subsequent gross dissection. **D**, TCGA pan-cancer database. **E**, COL1A1-related oncological survival analysis. **F**, Survival heatmap, selecting genes with the highest adverse prognostic confidence across different tumors and the COL1A1 gene. **G**, Analysis of COL1A1 protein expression in different tumor microenvironments (https://pdc.cancer.gov/pdc/browse). **H**, Representative immunohistochemical images from the Human Protein Atlas database (https://www.proteinatlas.org). I, Type I collagen-targeting peptides identified.


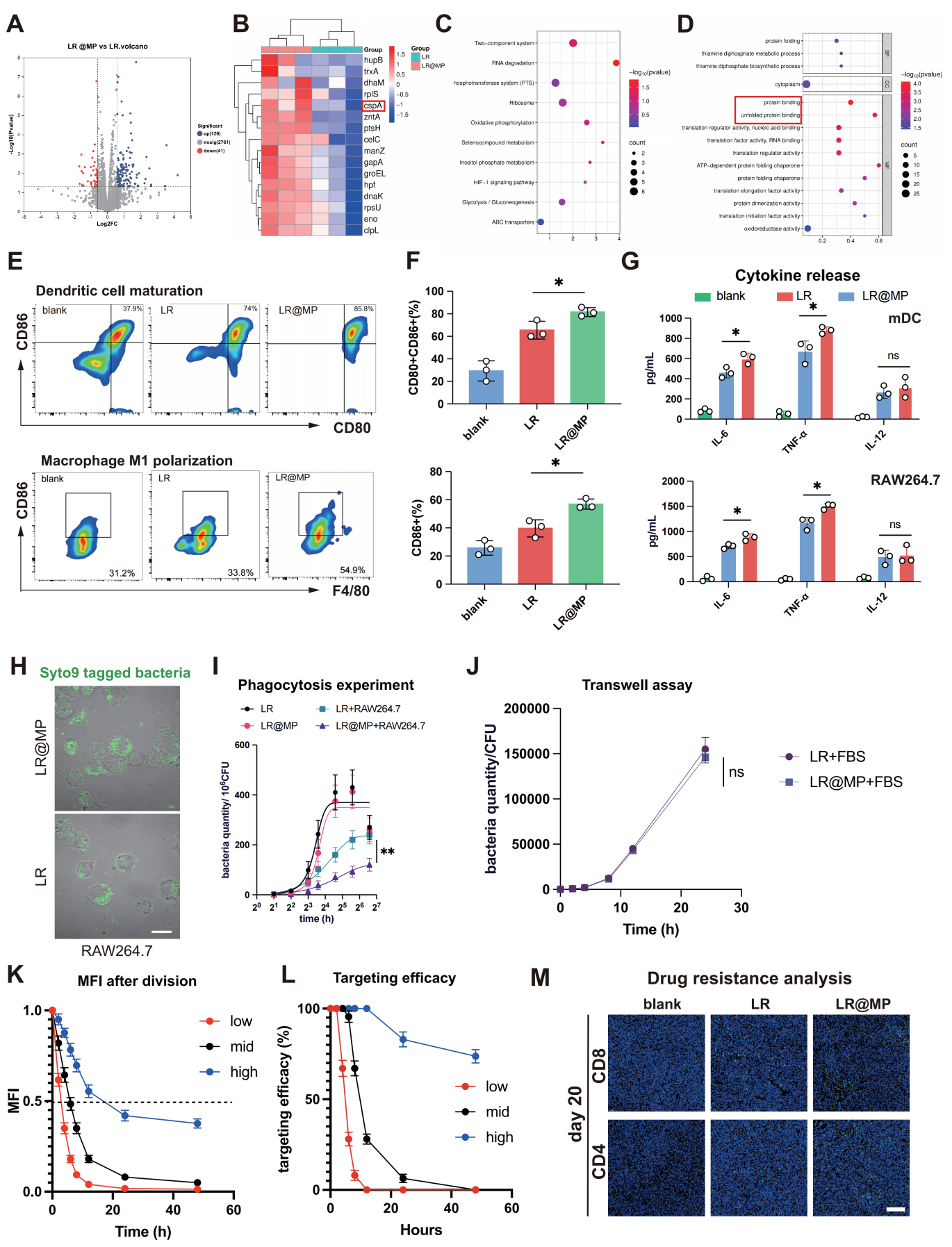


**Figure. S3** Construction and characterization of molecular pili-engineered probiotics. **A**, The overall distribution of regulated genes is represented by a volcano plot. **B,** Heat map top 16 genes, most of which are virulence-associated genes. **C**, The enrichment scatter plot to show KEGG enrichment analysis of a protein synthesis related KEGG terms. **D**, The enrichment scatter plot to show GO enrichment analysis of a protein synthesis related GO terms. **E, F,** Representative flow cytometry images and statistical data of RAW264.7 cells and BMDC cells activated in vitro (n = 3). **G**, Cytokine detection assay after bacterial stimulation of RAW264.7 and BMDCs (n=3). **H**, Representative confocal microscope images of cell phagocytosis of bacteria and RAW264.7. Scale bar, 40 μm. **I**, Phagocytosis experiments of macrophages at different time points (n=3). **J**, Transwell experiment: bacterial division was inhibited by mitomycin (n=3). **K**, Flow cytometry detection of MFI after different cell division times (n=3). Low, mid, and high represent different concentrations of bacteria. **L**, Targeting efficiency at different time pointsI (n=3). Targeting efficiency is the percentage of fluorescence intensity of bacteria adhering to the collagen culture plate relative to the total bacteria. **M**, Representative immunofluorescence (IF) images of tumor-infiltrating T cells. Scale bar, 150 μm.

**2. Experimental Section**

2.1. Bacterial culture

We purchased the bacteria (*Lactobacillus rhamnosus* (ATCC 53103), Escherichia coli Nissle 1917, Bifidobacterium longum (ATCC 15707)) from ATCC and cultured them on MRS or LB plates for 48 hours. Subsequently, ten clones were inoculated into fresh MRS medium and incubated for 12 hours at 37 °C, followed by collection through centrifugation at 3,000 rpm for a minimum of 10 minutes. The VPN20009 strain and Escherichia coli were kindly provided by Dr. Yang (IMM) and Dr. Chen (Ruijin Hospital). Prior to experimentation, bacterial growth curves were determined by measuring optical density (OD_600_).

**2.2. Synthesis of LR@MP**

First, obtain the probiotic LR with an OD_600_ value of 1, which was subsequently resuspend by Phosphate Buffered Saline (PBS), mixed with 0.2 mg/mL Sulfo-SMCC (Sigma). After 2 hours of standing still at 4 °C, the mixed system is added with MP or labbled MP (FITC-ahx-RLRELHLNNNCys). After at least one hour the obtain product was resuspend in PBS for characterization, or lyophilized for storage.The MP particles were custom-synthesized by Shanghai Apeptide Co., Ltd. (Shanghai, China).

**2.3. Proteomics and Phosphoproteomics Analysis**

LR and B16-F10 cells were co-cultured for 24 hours. Cells were then digested, and LR was removed via gradient centrifugation to retrieve the cell pellet. For all samples, the following procedures were sequentially performed: protein extraction, quality control, quantification, enzymatic digestion, desalting, peptide fractionation, and high-resolution mass spectrometry analysis in DDA (Data-Dependent Acquisition) mode. The qualitative and quantitative results obtained from database searching were subsequently subjected to bioinformatic analysis. The proteomic analyses were performed using the institutional core facility at Zhangjiang Campus of Fudan University.

**2.4. Characterization of LR and LR@MP**

Bacterial cell viability was assessed using a Cell Counting Kit-8 (BioAgrio) with a Multi-Detection Microplate Reader (BioTek, USA). Bacterial morphology was examined with a Hitachi SU8000 scanning electron microscope and an Olympus laser confocal microscope.

**2.5. Animals and cells**

Male C57BL/6 mice (6–8 weeks old) were housed in a specific pathogen-free (SPF) facility under controlled environmental conditions, including a temperature of 22 ± 2°C, relative humidity of 50 ± 10%, and a standard 12-h light/12-h dark cycle. The animals were provided with standard laboratory chow and water ad libitum. All animal experiments were performed in strict accordance with the Guide for the Care and Use of Laboratory Animals and were approved by the Institutional Animal Care and Use Committee (IACUC) of Chengxi Biotechnology (Approval No. CX052408088). To ensure animal welfare, humane endpoints were strictly enforced. Mice were euthanized via CO^2^ asphyxiation followed by cervical dislocation if the tumor volume exceeded 1,500 mm^3^, or if the mice exhibited significant clinical deterioration, defined as a body weight loss of >20% compared to baseline. The following cell lines were used in this study (RRIDs in parentheses): BxPC-3 (RRID:CVCL_0186), B16-F10 (RRID:CVCL_0159), MC-38 (RRID:CVCL_B288), Panc02 (Pan02, RRID:CVCL_D627), Hepa 1-6 (RRID:CVCL_0327), RenCa (RRID:CVCL_2174), HCT 116 (RRID: CVCL_0291), NIH-3T3 (RRID:CVCL_0594), MIA PaCa-2 (Mia, RRID: CVCL_0428) and HUV-EC-C (HUVEC, RRID:CVCL_2959). All cell lines were sourced from the Chinese Academy of Sciences. Prior to receipt and at key experimental checkpoints, all human cell lines underwent STR (Short Tandem Repeat) fingerprinting. For non-human cell lines (e.g., B16-F10, MC-38, Panc02, Hepa1-6, RenCa, and NIH-3T3), species-specific molecular authentication was performed: genomic DNA was extracted and subjected to species-specific PCR for the cell of origin, in parallel with a species-identification PCR. All cell cultures were maintained in Dulbecco’s Modified Eagle’s Medium (BioAgrio), supplemented with 10% fetal bovine serum (Gibco) and 1% penicillin-streptomycin (BioAgrio), under conditions of 37 °C and 5% CO_2_. BMDCs were isolated from the femurs and tibias of mice using standard techniques.

**2.6. *In vitro* flow cytometry**

The killing assay utilized the Zombie Aqua kit (Biolegend) to determine dead cells. BMDCs were stimulated with probiotics for 24 hours after 7 days of induction to maturation, while macrophages (RAW264.7) were directly stimulated with probiotics for 24 hours. The RAW264.7 and BMDCs exposed to bacteria were collected and subsequently resuspended in PBS. RAW264.7 cells were treated with anti-CD16/32, followed by staining with fluorophore-conjugated antibodies (Biolegend) F4/80 and CD86 for 30 minutes at room temperature prior to flow cytometry analysis using the BD LSR Fortessa X-20. In a similar manner, BMDCs were treated with CD11c, CD80, and CD86. Phagocytosis assays and immunofluorescence were evaluated using laser confocal microscopy (Olympus). For surface molecule detection, anti-PDL1 (‌ABclonal) and anti-H2-Kd (Invitrogen) were used to examine the expression of PD-L1 and MHC class I (H2-Kd) on mouse cells, while anti-Human PDL1 (‌ABclonal) and anti-HLA-ABC (Invitrogen) were employed to assess PD-L1 and MHC class I (HLA-ABC) expression on human tumor cells. All flow cytometry experiments were performed by first gating live cells selected with the Zombie Aqua kit before proceeding to subsequent analysis.

**2.7. Collagen-attach detection**

Bacteria were added to COL1-coated plates and incubated for 4 hours at 4 °C. After three washes, the plates were examined using a fluorescence microscope (Nikon). Fluorescence intensity was quantified with IMAGE J. The bacterial colonization ability on collagen was evaluated by assessing the adhesion of bacteria to type I collagen (derived from mouse tails).

**2.8. Western blotting**

Cell protein was quantified using a BCA assay kit and denatured by heating and using 5 × SDS loading buffer. Following gel electrophoresis and protein transfer, primary antibodies were applied, A goat anti-rabbit secondary antibody (Abcam) was utilized for detection in these blots. All primary antibodies were from Cell Signaling Technology (CST). Membrane protein detection does not require boiling.

**2.9. Live/dead assay**

The bacterial culture supernatant in DMEM was filtered through a 0.1 μm sterile filter, and then supplemented with 10% FBS for cell culture. For the attenuation method, the samples were fixed with paraformaldehyde for 15 minutes followed by thorough washing. For the killing assay, different cell lines were seeded in 96-well plates at a cell-to-bacteria ratio of 1:2 for cytotoxicity testing. After 12 hours, cell viability was assessed using a Cell Counting Kit-8 (BioAgrio). For the gradient killing assay, various cell lines were plated in 6-well plates and tested at different cell-to-bacteria ratios. After 12 hours, all cells were harvested and subjected to live/dead cell staining using the Zombie Aqua kit. To further validate cell viability, Calcein-AM/PI staining was performed (Beyotime, China). For the induction experiment, a cell-to-bacteria ratio of 1:2 was used. The medium was replaced every 24 hours, removing dead cells and free probiotics, followed by the addition of a small amount of fresh probiotics. For the OT-1 and OVA cytotoxicity assay, the experiment was conducted according to established protocols, with a fixed effector-to-target cell ratio of 10:1 between OT-1 cells and tumor cells.

**2.10. Mitochondrial detection**

After incubation with Annexin V/PI (Biolegend), cell apoptosis was assessed using flow cytometry (BD LSR Fortessa X-20). Mitochondrial membrane potential was evaluated after approximately 12 hours with JC-1 Mitochondrial Membrane Potential Kits (Beyotime, China) using a confocal microscope (Olympus). ATP levels were measured with ATP kits (Beyotime, China). Reactive oxygen species (ROS) were detected using a ROS assay kit (Sigma) and visualized under a laser confocal microscope (Olympus). Mitochondrial ROS was detected using the MitoSOX Red kit (Invitrogen) and visualized by confocal microscopy (Olympus).Intracellular calcium ions were measured with the Fluo-4 AM assay kit (Abcam).

**2.11. immunofluorescence assay**

For the immunofluorescence assay, after permeabilization and fixation, cells were incubated with either anti-p65 (CST) or anti-HIF-1α antibody (CST) for 1 hour, followed by incubation with a fluorophore-conjugated secondary antibody for another hour. After DAPI counterstaining, samples were visualized using confocal microscopy (Olympus).

**2.12. *In vivo* tumour treatment**

Tumor volume (TV) was measured using calipers and calculated using the formula: width × width × length × 0.5. Ascitic fluid was obtained through abdominal puncture, and samples were collected for Wright staining. In the short-term treatment trial, all mice were retained. In the long-term treatment trial, mice with tumor volumes exceeding ethical limits were automatically excluded and not included in the experimental analysis. For transcriptomic analysis of melanoma tissues, total RNA was extracted using TRIzol reagent (Invitrogen) followed by isopropanol precipitation and ethanol washes. After library construction and quantification (VAHTS Universal V8 RNA-seq Library Prep Kit for Illumina, Qubit4.0), paired-end sequencing was performed on the NovaSeq 6000 platform (Illumina).

**2.13. *In vivo* flow cytometry**

Tumor tissues were collected and homogenized in cold PBS buffer with the addition of digestive enzymes to create single-cell suspensions. The cells were initially incubated with anti-CD16/32 and subsequently stained with fluorophore-conjugated antibodies (Biolegend) including CD45, CD3, CD4, CD8, CD11c, CD80, CD86 and Foxp3 for 60 minutes at room temperature prior to flow cytometry analysis (BD LSR Fortessa X-20). Staining for Foxp3 was performed after the cells were fixed and permeabilized using Fixation Diluent and Permeabilization Diluent. All flow cytometry experiments were performed by first gating live cells selected with the Zombie Aqua kit before proceeding to subsequent analysis. The antibody staining procedure was performed strictly following the manufacturer's instructions.

**2.14. Histopathological examination**

The tissue was fixed with 4% paraformaldehyde, dehydrated, and then sequentially embedded in paraffin. Prepare tissue slices for H&E staining. For immunofluorescence, we first performed deparaffinization and antigen retrieval. Subsequently, 5% BSA was used to block non-specific binding sites on the sections. Anti-CD4 and anti-CD8 antibodies (1 : 300 dilution) were then added and incubated for 30 minutes. After three washes with PBS, the sections were incubated in the dark with anti-Rabbit IgG secondary antibody (1 : 500 dilution) at 37 °C for 30 minutes. Following three additional washes, all sections were stained with DAPI and examined using a confocal microscope (Nikon).

**2.15. Intracellular calcium measurement**

To investigate the source of calcium overload and its role in LR-mediated cytotoxicity, calcium chelation experiments were performed using the extracellular calcium chelator EGTA (Sigma-Aldrich) and the intracellular calcium chelator BAPTA-AM (Thermo Fisher Scientific). B16F10 cells were seeded in 96-well plates (5×10^3^ cells/well) or 6-well plates (2×10^5^ cells/well) and allowed to adhere overnight. Prior to bacterial infection, cells were pre-treated with EGTA (2 mM) or BAPTA-AM (10 μM) for 1 hour at 37°C. Following pre-treatment, cells were co-cultured with LR (E:T=10:1) in the continued presence of the chelators. For calcium-free conditions, cells were washed and cultured in calcium-free DMEM (Gibco) during the infection period. After 12 hours of co-culture, cell viability was assessed using the CCK-8 assay, and mitochondrial ROS levels were quantified via flow cytometry as described above. To validate the impact on the electron transport chain, the enzymatic activities of Complex I, III and V were also measured in the presence of EGTA as described in the respective enzymatic assay sections (Cayman Chemical).

**2.16 Bacterial motility and chemotaxis assay**

To evaluate whether surface modification affects bacterial motility, a transmembrane migration assay was performed using Transwell chambers (Corning, 8.0 μm pore size). Briefly, LR and LR@MP were resuspended in serum-free DMEM to an optical density (OD_600_) of 0.5. 200 μL bacterial suspension was added to the upper chamber. To create a chemotactic gradient, the lower chamber was filled with 600 μL of DMEM supplemented with 10% FBS (as a chemoattractant) or serum-free DMEM (as a negative control). The chambers were incubated at 37°C for 4 hours. Subsequently, the media from the lower chamber containing the migrated bacteria was collected. The number of migrated bacteria was quantified directly under a light microscope using a hemocytometer.

**2.17 Time-dependent phagocytosis**

To investigate the phagocytic kinetics of macrophages towards the engineered bacteria, a time-course phagocytosis assay was conducted using the standard colony-forming unit (CFU) counting method. RAW264.7 macrophage cells were seeded in 12-well plates (1×10^5^ cells/well) and allowed to adhere overnight. The cells were then co-cultured with LR or LR@MP at a multiplicity of infection (MOI) of 10 at 37°C. At predetermined time points, the cells were washed three times with PBS to remove non-adherent bacteria. To eliminate extracellular bacteria that were attached but not internalized, the cells were incubated with DMEM containing gentamicin (200 μg/mL) for 1 hour. Subsequently,the cells were washed thoroughly and lysed with 0.1% Triton X-100 in PBS for 10 min to release intracellular bacteria.The lysates were serially diluted and plated onto MRS agar plates. After 24 hours of incubation at 37°C under anaerobic conditions, the number of CFUs was counted to quantify the viable intracellular bacterial load.

**2.18 Evaluation of Surface Coating Retention during Bacterial Division**

To assess the stability and dilution kinetics of the surface modification during bacterial proliferation, flow cytometry was employed. LR was conjugated with FITC-labeled MP (LR@MP-FITC) as described above. The initial bacterial suspension was inoculated into fresh MRS broth at (LOW: OD_600_=0.1, MID: OD_600_=0.2, HIGH: OD_600_=0.4) and cultured at 37°C. At predetermined time points corresponding to different bacterial generations, aliquots were collected and washed twice with PBS to remove free peptides. The retention of the surface coating was quantified by measuring the percentage of FITC-positive bacteria and the Mean Fluorescence Intensity (MFI) using a flow cytometer (Beckman Coulter). The "functional threshold" of the coating was determined as the time point at which the fluorescence intensity dropped below a significant detection level relative to the baseline.

**4.19. Statistical analysis**

The results presented in this article are expressed as mean values ± standard deviation (s.d.), as detailed in the corresponding Figure legends. Statistical analyses were performed using GraphPad Prism software version 10. For comparisons between two groups, a two-sided unpaired Student’s t-test was used. For comparisons among multiple groups, one-way analysis of variance (ANOVA) was performed followed by Tukey’s post-hoc test for multiple comparisons. For time-dependent data (e.g., tumor growth curves), two-way ANOVA was utilized followed by Bonferroni’s multiple comparisons test. Survival analysis was performed using the Kaplan-Meier method, and statistical significance was determined by the Log-rank (Mantel-Cox) test. A P-value of less than 0.05 was considered statistically significant. Significance levels are indicated in the figures as follows: ∗ P<0.05, ∗∗ P<0.01, ∗∗∗P<0.001, and ∗∗∗∗ P<0.0001. We used DESeq2 (R version 3.6.2) to analyze gene expression differences. Differentially expressed genes with an FDR less than 0.05 and a fold change≥2were subsequently used for GO and KEGG enrichment analysis.
